# Occurrence of Second Oral Potentially Malignant Disorder following Excision of Primary Lesion: A Prospective Study of Cases from a Tertiary Care Centre

**DOI:** 10.1007/s12663-022-01764-9

**Published:** 2022-07-20

**Authors:** Adarsh Kudva, Mathangi Kumar, Evit Rajan John, Vasantha Dhara

**Affiliations:** 1 Department of Oral and Maxillofacial Surgery, Manipal College of Dental Sciences, Manipal, Manipal Academy of Higher Education, Manipal, Karnataka 576104 India; 2 Department of Oral Medicine & Radiology, Manipal College of Dental Sciences, Manipal, Manipal Academy of Higher Education, Manipal, Karnataka 576104 India; 3 School of Public Health, UT Health Science Center Houston, 7000 Fannin St #1200, Houston, TX 77030 USA

**Keywords:** Oral cancer, Smoking, Recurrence, Intervention, Oral dysplasia

## Abstract

**Background:**

Early diagnosis and timely management of potentially malignant oral disorders may prevent malignant transformation and prompt diagnosis of frank malignancies favours better prognosis. The aim of this study was to evaluate the outcome of surgical management of oral potentially malignant disorders of the oral cavity and observe the prevalence of recurrence at the primary site and occurrence of another potentially malignant lesion in these patients.

**Methods:**

The study participants included patients who had undergone clinical oral examination, surgical excision of biopsy-proven cases of dysplastic oral potentially malignant disorders (leukoplakia, erythroplakia, non-healing ulcerative and erosive areas, etc.) who were on routine follow-up as per the standard guidelines. These patients were followed up closely during each monthly follow-up visit for the first year. The patients were then prospectively analysed for any recurrence of lesion. On follow-up visits, detailed clinical oral examination was done to note the prevalence of a new lesion in any oral cavity sub site other than the previous site. If a new lesion was detected, then biopsy followed by surgical excision was followed as per standard guidelines. The follow-up period after the second surgical intervention was 12 months.

**Results:**

Fifty patients with potentially malignant oral disorders underwent surgical excision. The majority of the study subjects were males (39/50) and 41 of them were below 65 years of age. Of 50 patients, 13 (26%) had second oral potentially malignant lesion other than the primary site. The rate of recurrence of the lesions at the primary site was 4% (2/50). Of these patients with recurrence, all had malignant transformation (2/2). Also, patients who were initially diagnosed with moderate dysplasia had a higher chance of recurrence. A second lesion at a site different from the primary lesion was seen in 26% of the cases.

**Conclusion:**

Surgical management of such lesions with one-centimetre oncological margins in all dimensions contrary to the routine five millimetre surgical margins reduces the chance of recurrence.

## Introduction

Oral cancer contributes to one-third of all cancers in India, and data from GLOBOCAN 2020 suggest that cancers of the lip and oral cavity are highly frequent in Southern Asia and the leading cause of cancer death among men especially in India and Sri Lanka [1]. Oral potentially malignant disorders (OPMD) are defined as ‘the risk of malignancy being present in a lesion or condition either during the time of initial diagnosis or at a future date’ [2]. ‘Potentially Premalignant Oral Epithelial Lesions’ (PPOELs) harbour histological changes such as squamous hyperplasia, mild dysplasia, moderate dysplasia, sever dysplasia and carcinoma in situ [3]. Ho PS reported a malignant transformation rate of 7.62 per 100 persons-year in males. Early diagnosis and timely treatment of these lesions may prevent malignant transformation and timely diagnosis of frank malignancies would favour better prognosis in terms of morbidity and quality of life [4].

The concept of ‘Field Cancerisation’ by Slaughter states that the presence of a clinically detectable premalignant lesion(s) due to prolonged exposure to a carcinogen increases the risk of it happening in other sites in the oral cavity due to ‘field effect’ [5]. This effect describes the occurrence of lesions at multiple sites and the occurrence of a second primary lesion even after the management of the primary lesion [6]. It also explains the high rate of malignant transformation at high-risk sites (tongue and floor of the mouth) and the presence of subclinical lesions elsewhere. This necessitates the need for long-term follow-up for any patient with a history of dysplasia as they may develop lesions in other intraoral sites [7]. The process of carcinogenesis initiates from multiple genetic and epigenetic alterations in the mucosa which can lead to the clonal expansion of premalignant daughter cells in a particular field. The genetically altered stem cells form a clonal unit comprising daughter cells from which the patch expands into the adjacent areas in subsequent steps following further modifications. This triggers sequential cellular transformations that ultimately lead to the replacement of the normal epithelium by a proliferating field. However, there is a population of cells with early genetic changes, which does not demonstrate any histological alterations, thus explaining the concept of field cancerization [8, 9].

The aim of this study was to evaluate the outcome of surgical management of oral potentially malignant disorders (OPMD) and observe the incidence of metachronous recurrence of OPMD at primary site and occurrence of another (new/ second) OPMD in these patients. Also, to determine the various factors (age, gender, habit history) that can lead to the recurrence of second OPMD, thereby strengthening the concept of field cancerization.

## Materials and Methods

This cross-sectional prospective study included patients who reported to the tertiary care centre from 2014 to 2018 with oral potentially malignant lesions. This study was approved by the Institutional Ethics Committee (IEC 173/2018). The study participants included patients who had undergone clinical oral examination in Department of Oral Medicine and Radiology and surgical excision and biopsy-proven cases of dysplastic OPMD lesions (leukoplakia, erythroplakia, non-healing ulcerative and erosive areas, etc.) in the Department of Oral and Maxillofacial Surgery who were on routine follow-up as per the standard guidelines. These patients were followed up closely with monthly follow-up visit during the first year, three monthly for the second year, 6 monthly for the third year and annual follow-up for the following year. The data regarding the age and sex, oral abusive habit history, life style practices and site of primary potentially malignant oral lesion were recorded for each patient. The patients were then prospectively analysed for any recurrence of new (second) OPMD lesion in any other sub site in the oral cavity other than the previous site. Second OPMD refers to the incidence of metachronous recurrence of OPMD at the primary site or another new site. If a new lesion was detected, then the patient would be taken up for surgical removal of the lesion according to the protocol.

**Fig. 1:**
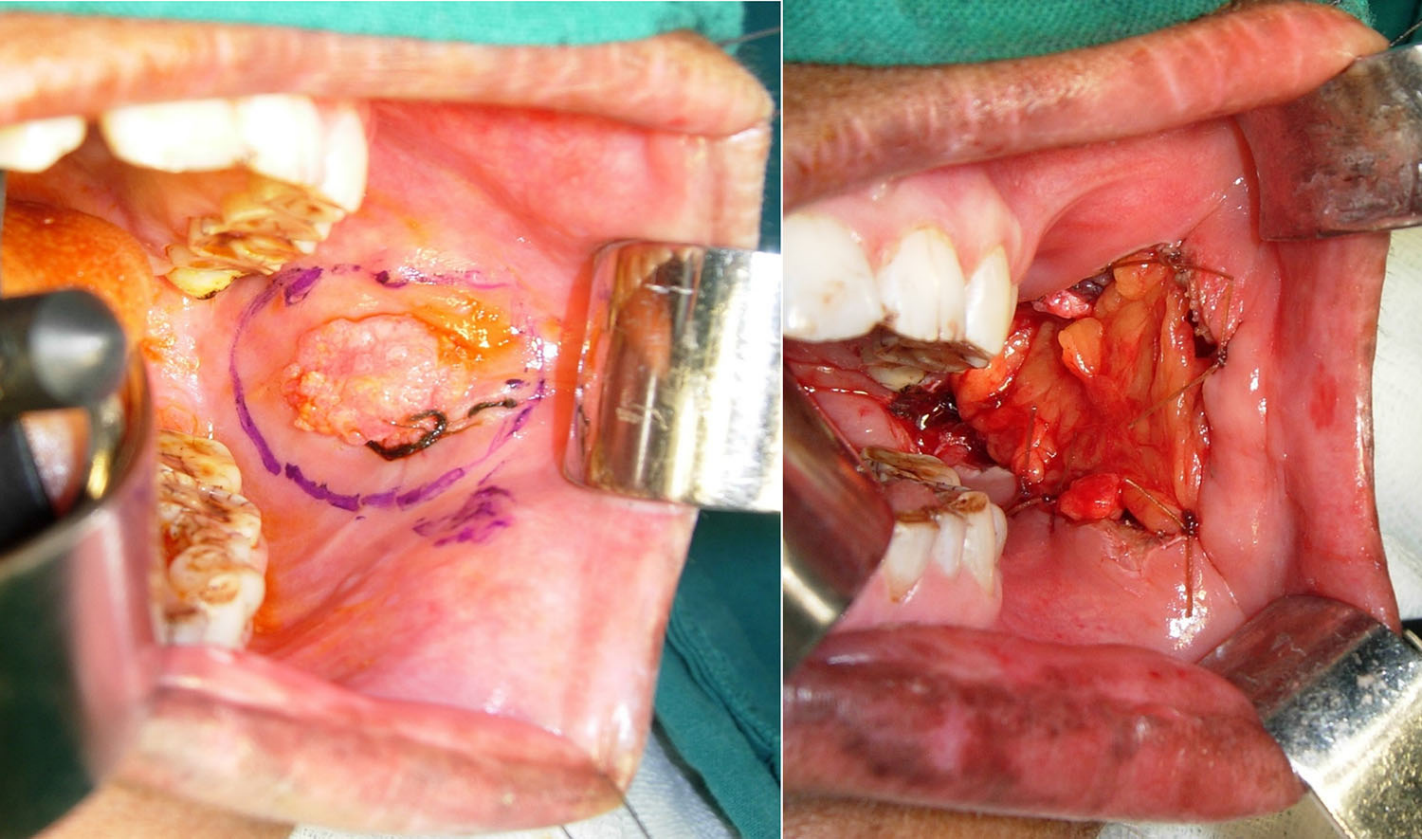
Photograph depicting **a** one-centimetre oncological safe margin for excision of the lesion and **b** reconstruction of the surgical site with local flaps (buccal fat pad)

The surgical procedure employed was wide local excision of lesion under local anaesthesia with 1 cm oncological margins in all three dimensions. Reconstruction of the surgical defect was done using collagen membrane, platelet rich fibrin membrane, local mucosal flaps, buccal pad fat and nasolabial flaps as applicable for the particular case (Fig. 1). The surgically excised lesions were sent for histopathological examination and the final diagnosis directed definitive management of the patients according to standard oncological principles. At every follow-up visit, all the patients were reinforced about the importance of cessation of oral abusive habit and they were advised to follow healthy diet and lifestyle.

The collected data were entered in Microsoft Excel sheet and descriptive statistics was gathered from the obtained data.

## Results

Fifty patients with oral potentially malignant disorders underwent surgical excision during the study period (2014 to 2018). There were 39 males and 11 females of which 82% of them were less than 65 years of age. Buccal mucosa was the most common site of the OPMD (48%). Other sites of OPMD were tongue (30%) > lip (12%) > alveolus (4%). The most commonly operated lesions were from buccal mucosa (48%) followed by lateral border of tongue (30%). However, the occurrence of a second lesion from these sites were 25% and 33% respectively. Other sites of excision included lip (20%), palate (2%), gingivobuccal sulcus (2%), alveolus (4%) and faucial pillar (2%). The detailed demographic data of the included patients are given in Table 1. The most common type of dysplatic lesion noted in the present study was leukoplakia (58%). 21 subjects had the history of smoking tobacco (cigarette, beedi) and 22 subjects had smokeless tobacco habit. Also, seven individuals reported of having dual habits (smokeless and smoking form). Seven subjects did not report of any oral abusive habit. The rate of recurrence of the lesions at the primary site was 4% (2/50), i.e. recurrence of premalignant lesion at the same site as that of the primary lesion. Two out of fifty patients (4%) had malignant transformation. Of these patients with recurrence, all had malignant transformation (2/2). Of the 4 patients who exhibited micro-invasion at the primary site, two of them had malignant transformation. The site-wise distribution of the recurrent lesions in these patients is depicted in Table 2.

**Table 1:** Demographic data of patients prior to surgery

Variable	Number (%)
*Sex*	
Female	11 (22)
Male	39 (78)
*Mean age (SD)*	
65 or less	41 (82)
Over 65	9 (18)
*Site*	
Buccal	24 (48)
Lip	6 (12)
Tongue	15 (30)
Retromolar	0 (0)
Floor of mouth	0 (0)
Palate	1 (2)
GB Sulcus	1 (2)
Alveolus	2 (4)
Faucial Pillar	1 (2)
*Drinking alcohol*	
Yes	19 (38)
No	31 (62)
*Smoke tobacco*	
Cigarette	13 (26)
Beedi	8 (16)
*Smokeless tobacco*	
Arecanut	10 (28)
Gutka	12 (46)
*Type of lesion*	
Leukoplakia	29(58)
Erythroplakia	12(24)
Non-healing ulcer	3(6)
Erosive lesions (erosive lichen planus, speckled lesions)	6(12)
*Histopathology prior to surgery*	
No dysplasia	0 (0)
Mild dysplasia	20 (40)
Moderate dysplasia	18 (36)
Severe dysplasia	8 (16)
Micro-invasive	4 (8)

**Table 2:** Recurrence based on site

Site	Recurrence
Buccal	6 Recurrences in 24 primary lesions
Lip	3 Recurrence in 6
Tongue	5 Recurrences in 15
Retromolar	0 Recurrence in 0
Floor of mouth	0 Recurrence in 0
Palate	1 Recurrence in 1
GB Sulcus	1 Recurrence in 1
Alveolus	2 Recurrence in 2 cases
Faucial Pillar	0 Recurrence in 1

The overall occurrence of a new potentially malignant lesion (other than the previous site) was noted in 26% (13/50) of the individuals. Of these new lesions, 11/13 cases showed mild dysplasia and 2/13 had moderate dysplasia. The histopathological characteristics of primary lesions that had a new OPMD at a second site were 1 case with mild dysplasia, 7 cases with moderate dysplasia and 5 cases had severe dysplasia. The recurrence based on type of primary lesions observed is shown in Table 3.

**Table 3:** Recurrence based on type of primary lesions

Primary lesion dysplastic feature	No dysplasia	Mild	Moderate	Severe	Malignancy
Mild Dysplasia (20)	2	1	0	0	0
Moderate (18)	2	5	0	1	1
Severe Dysplasia (8)	0	1	1	0	1
Micro-invasive SCC (5)	0	0	1	0	1

Also it was observed that patients who had dual habit history of tobacco chewing (arecanut/ gutka) along with smoking habit (beedi/cigarette/cigar) had the highest rate of a second lesion (6 lesions in 6 patients) (Table 4).

**Table 4:** Habit history and its association with recurrence

Smoking tobacco	3 Recurrence in 22 patients
Smokeless tobacco	6 Recurrence in 37 patients
Smoke and smokeless (Dual habit)	6 Recurrence in 6 patients
No smoke or smokeless (No habits)	0 Recurrence in 7 patients

Also, those who were initially diagnosed with moderate dysplasia (18/50 patients) had a higher chance of recurrence. It was also observed that, compared to patients with a single habit, those who had a dual habit had a higher chance of recurrence.

## Discussion

It is well recognized that the progression of a potentially malignant lesion to frank oral squamous cell carcinoma is not a singular event, but a gradual process of morphologic, genetic and histological aberrations that lead to malignant transformation. The occurrence of two or more lesions in a patient at the same time is termed as synchronous lesion, whereas lesion occurring after a three-month post-op period is called as a metachronous lesion [10].

In the present study, it was noted more males (78%) present with OPMD than females (22%) with buccal mucosa being the most commonly affected primary site. The most commonly noted OPMD in the present study was homogenous leukoplakia. Fernanda Weber Mello et al. [11]. performed a systematic review on prevalence of oral potentially malignant disorders worldwide and noted that the most common OPMD was oral submucous fibrosis (4.96%) and males were affected [11]. In this study, a majority of patients presented with mild (40%) and moderate dysplasia (36%).

Speight M et al. evaluated the risk factors that favour the progression to OPMD to malignancy and stated that factors like sex; site and type of lesion; habits, such as smoking and alcohol consumption; and the presence of epithelial dysplasia on histological examination dictated the transformation to malignancy [10]. In the present study, buccal mucosa was the affected site by the second OPMD following excision of primary lesion. Second OPMD refers to the incidence of metachronous recurrence of OPMD at the primary site or another new site. Patients with smoking tobacco habit exhibited the maximum occurrence of a second OPMD compared to smokeless tobacco (50%). However, findings by Jani et al. suggest that tobacco consumption in any form is hazardous and causes various kinds of oral premalignant lesions and people with exposure to tobacco are 43.62 times at higher risk of developing OPMD in the oral cavity [12].

The overall prevalence of a second OPMD other than the primary site was 26% in the present study; ie.13/50 patients (26%) had second oral potentially malignant lesion other than the primary site. Studies evaluating the malignant transformation rates of OPMD have been performed, but there is no literature available that describes the occurrence of second OPMD following excision of primary lesion. The occurrence of OPMD at another site can be explained by the concept of field cancerization. Repeated and constant exposure of carcinogens to the aerodigestive tract will predispose the individual to develop isolated or multiple-site lesions as described by Slaughter [5]. The concept of field cancerization proposes that the normal tissue adjacent to neoplastic region contains genetic markers which can, overtime, lead to recurrence of a second primary lesion [5, 13].

Thomson et al. performed a retrospective clinico‐pathological analysis on 590 patients with oral potentially malignant disorders. It was observed that (16.8%) developed cancer, 67.9% of lesions arose at new sites, 42.3% of cases showed micro‐invasive arising from severely dysplastic precursors. The most common sites of recurrence of the lesions was the ventro‐lateral tongue and the floor of mouth [14]. However in the present study, 4/50 (8%) of the cases showed micro-invasion out of which half of them (2 out of 4 patients) showed malignant transformation. The most common site of the second OPML lesion in the present study was the buccal mucosa.

Systematic review on potentially malignant disorders and dysplasia by Iocca O et al. showed that moderate/ severe dysplasia bears a much higher risk of cancer evolution than mild dysplasia [15]. These findings were consistent with the observations from the present study where 50% of the individuals with moderate dysplasia developed a second OPMD at another site.

In the present study, buccal mucosa exhibited the maximum new OPMDs. Pei-Shan Ho et al. conducted a retrospective cohort study on malignant transformation of oral potentially malignant disorders in males and observed that the rate of transformation was highest in subjects diagnosed with oral epithelial dysplasia with tongue being 2.41 times at more risk when compared with buccal lesions [16]. In the present study sites like the tongue (*n* = 1), the hard palate (*n* = 1) and alveolar mucosa (*n* = 1) showed malignant transformation.

While it is impossible to predict the behaviour of premalignant lesions on the basis of clinical appearance, histopathological features or predict the occurrence of a second OPMD, it is well evident that routine and aggressive follow-up protocols is earliest way for prompt detection of such lesions and treat it with minimum morbidity. This ensures secondary level of prevention of these lesions and its malignant transformation. Patients have to be counselled for their oral abusive habit which can significantly reduce the need for aggressive pharmacotherapy and significantly decrease the risk of transformation [17, 18].

In the present study, wide local excision of all lesions with one-centimetre margins in all dimensions was performed which is unlike the routinely recommended 5 mm margins for potentially malignant oral disorders. We postulate that resecting these lesions with adequate margins and reconstructing with local flaps reduces the chance of recurrence. The rate of recurrence of the lesions at the primary site was 4% (2/50 patients). Also, if the histopathological report is suggestive of malignancy/micro-invasion, this approach eliminates the need for re-excision of lesion. Regular self-evaluation and self-oral examination along with professional evaluation allows for early diagnosis of recurrent lesions.

## Conclusions

In conclusion, the present study suggests that surgical management of such lesions with one-centimetre oncological margins in all dimensions contrary to the routine five millimetre surgical margins reduces the chance of recurrence. Currently there are no standardized protocols for follow-up of patients with potentially malignant oral lesions or history of excision of such lesions; but there can be tailor-made according to the patient factors and risk stratification. Time for malignant transformation of a potentially malignant oral lesion is unpredictable and varies from months to years and from patient to patient. Hence, these patients must be on strict long-term follow-up so that new lesions may be readily diagnosed.

## Data Availability

Yes.
